# Resurgence of yellow fever in Africa in 2022: a glance on protective measures

**DOI:** 10.1097/JS9.0000000000000117

**Published:** 2023-02-16

**Authors:** Hitesh Chopra, Neil Patel, Yashendra Sethi, Talha B. Emran

**Affiliations:** a Chitkara College of Pharmacy, Chitkara University, Rajpura, Punjab; b GMERS Medical College Himmatnagar, Himmatnagar, Gujarat; c Government Doon Medical College, HNB Medical Education University, Dehradun, Uttarakhand, India; d Department of Pharmacy, BGC Trust University Bangladesh, Chittagong; e Department of Pharmacy, Faculty of Allied Health Sciences, Daffodil International University, Dhaka, Bangladesh

Yellow fever is a flaviviral disease endemic in tropical areas of South America and sub-Saharan Africa. It is borne by mosquitoes of the genera *Haemagogus* and *Aedes* (*Stegomyia*) and maintained in nature by the transmission cycle between these mosquitoes and nonhuman primates. The yellow fever virus most often infects humans and monkeys. Mosquitoes transmit the virus from monkeys to humans. Following transmission from an infected human or monkey host, the yellow fever virus travels through the mosquito’s circulatory system before resting in its salivary glands. The virus is spread when an infected sylvatic mosquito bites a susceptible nonprimate host, such as a monkey (jungle yellow fever); or it spreads interhumanly through *Aedes aegypti*, which breeds in water-containing vessels, seen more commonly in developed areas (urban yellow fever)^1^.

The incubation period for yellow fever is between 3 and 6 days. Most patients present with symptoms of headache, fever, chills, myalgia, and nausea (phase 1) and report improvement after 3 or 4 days (phase 2). However, in addition to liver and renal issues, a return of symptoms has been reported by a subset of individuals (phase 3). The phase is marked by the return of high-grade fever and is characterized by jaundice, black urine, abdominal discomfort, and vomiting (Fig. 1)^2^.

**Figure 1:**
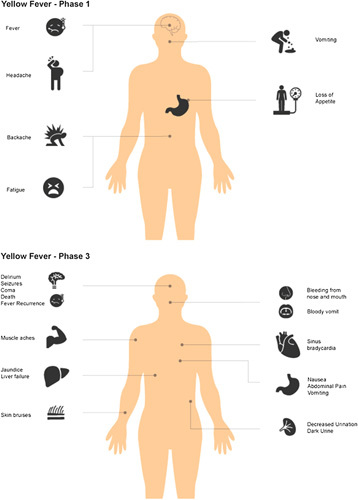
Symptoms in different phases of yellow fever.

Yellow fever outbreaks have been seen to occur if the following three conditions are fulfilled: introduction of the virus into a new cluster of nonimmune human primates, presence of anthropophilic vectors, and insufficiency of preventive measures^3^. Those visiting yellow fever-endemic areas could spread the disease to nations where it is not endemic. Many nations demand a yellow fever vaccination certificate before entry, especially from those who reside in or travel to yellow fever-endemic regions. In the World Health Organization (WHO) African Region, yellow fever is one of the top five most reported incidents in both 2019 and 2020. The worldwide plan to eradicate yellow fever epidemics has identified 27 nations in Africa as high-risk countries in terms of the time and intensity of yellow fever virus transmission, transmission potential, and evaluation of urban risk. Complex viral transmission continues in the area, with 184 confirmed cases and 274 probable cases reported by 12 nations between January 1, 2021, and August 26, 2022^4^. This includes 21 fatalities. The emergence or reemergence of vector-borne viral diseases is multifactorial. In 2020, there were reportedly two new cases of yellow fever in vaccinated West African nations (in Guinea and Senegal, now contained). Twelve nations in the area have reported both possible and confirmed cases since late 2021.

Increased morbidity and death have occurred from the ongoing multicountry epidemics and active viral circulation in West, Central, and East Africa. Vaccination rates against yellow fever have been below par in many of the worst-affected nations and subsets of the population. Yellow fever immunization coverage among African children was predicted to be 47% in 2021 by the WHO and the United Nations Children’s Fund^5^. This is much below the threshold of 80% needed for herd immunity against yellow fever; a huge population is still at risk of contracting the disease, and its transmission is likely to continue.

The prevention strategies mainly include vaccination, vector control, and epidemic preparedness and response. An inexpensive and secure vaccine exists to protect against yellow fever; one shot provides lifelong immunity. The vaccine does not need a booster shot. By 10 days, 80–100% of vaccinated individuals develop protective immunity, and more than 99% develop immunity within 30 days. In the past, *A. aegypti* – the urban yellow fever vector – was effectively eradicated from most of Central and South America by widespread mosquito control programs, but it has again recolonized these areas’ cities. The vectors have developed resistance to commonly used insecticides, and several pesticides have been removed from the market due to safety concerns, which makes developing newer, safe, and efficient insecticides a challenge. The WHO advocates preparedness for possible outbreaks, and thus developing tools for early detection and being prepared for a rapid response by emergency vaccination is essential^6^.

Quantifying the risk of spread by viremic travelers with the potential to propagate local transmission is imperative to assessing the risk of global yellow fever spread^7^.

Identifying the potential breeding areas of these mosquitoes may help officials target their efforts to improve surveillance and monitoring and identify potential locations for vector control initiatives. With the aim to limit spread to neighboring countries the WHO urges thorough monitoring with strong cross-border cooperation and information sharing. Consequentially, phylogenetic and phylogeographic tools can be utilized to understand outbreaks and develop countermeasures^8^.

## Ethical approval

Not applicable.

## Sources of funding

No funding.

## Author contributions

H.C., N.P., and Y.S.: conceptualization, writing – original draft preparation, writing – reviewing and editing. T.B.E.: conceptualization, writing – reviewing and editing, visualization.

## Conflicts of interest disclosure

The authors declare that they have no financial conflict of interest with regard to the content of this report.

## Research registration unique identifying number (UIN)

None.

## Guarantor

Talha Bin Emran, Department of Pharmacy, BGC Trust University Bangladesh, Chittagong 4381, Bangladesh. Tel: +880 303 356 193, Fax: +880 312 550 224. https://orcid.org/0000-0003-3188-2272.

## Provenance and peer review

Not commissioned, internally peer-reviewed.

## Data statement

No specific data collected for the above manuscript.
